# Differential expression of micro RNA-29 family in non-diabetic adults of diabetic and non-diabetic parents

**DOI:** 10.1186/s13104-021-05703-8

**Published:** 2021-07-28

**Authors:** Uzair Abbas, Bushra Imdad, Sikander Adil Mughal, Israr Ahmed Baloch, Afshan Mehboob Khan, Durr-e-Sameen Kamran

**Affiliations:** 1 Present Address: Department of Physiology, Dow University of Health Sciences, Karachi, Pakistan; 2 Department of Education and Research Mayo Clinic, Jacksonville, FL USA; 3 Department of Pathology, Dow University of Health Sciences, Karachi, Pakistan

**Keywords:** Diabetes mellitus, Early diagnosis, Biomarker, Micro RNA

## Abstract

**Objective:**

MicroRNAs are known to regulate 60% of genes at post translational level. MicroRNAs including Micro RNA-29 family play a vital role in cellular activities and have validate role in numerous metabolic disorders inclusive of diabetes mellitus and its complications. While micro RNA profile changes years before the occurrence of disease. This cross-sectional study was conducted in non-diabetic adults of diabetic and non-diabetic parents to explore the early changes in expression of micro RNA-29 family as it can be served as early biomarker of type 2 diabetes in non-diabetic adults. This study was conducted from January 2019 to January 2021. Micro RNA was extracted from plasma of 50 participants and expression was compared through qPCR. While data was analyzed through SPSS version 21.0.

**Results:**

29a and 29b had lower expression in participants with family history of DM compared to those having no family history of DM (P < 0.0001). While micro RNA 29c was found to be significantly higher in participants with positive family history of type 2 diabetes as compared to those without family history of diabetes (P = 0.001)**.**

**Supplementary Information:**

The online version contains supplementary material available at 10.1186/s13104-021-05703-8.

## Introduction

Diabetes Mellitus is a complex and multi factorial disease which is caused either by insufficient production of insulin or insulin resistance by the cells or both [1]. Presently there are more than 500 million prevalent cases of type 2 diabetes across the world [2]. In Pakistan, type 2 DM is 16.98% prevalent [3]. Early diagnosis of the diabetes holds an immense importance because if it is not diagnosed timely than it may have very unsound effects on population leading to higher rates of hospitalizations due to the related complications which are dangerous and widely affect the quality of life even early death [4].

The miRNA-29 group has been known to become important regulator of glucose metabolism and has altered expression in diabetics due to its different targets [5–9]. So, it is supposed to be important in studying diabetes and its related micro and macro vascular complications. Researches have reported up regulation of MiR-29 in target insulin tissues of animal models and in serum of diabetic patients along with diabetic risks such as retinopathy, kidney disease and cardiovascular disorders. Since the discovery of micro-RNA multiple researches have been directed to correlate the role of micro RNAs in diabetes and its associated risks. MiR-29 knockout mice were observed having high fasting glucose and impaired serum insulin release [5]. MiR-29 was also increased by two folds in hepatocytes of diabetic mouse indicate negative regulation of gluconeogenesis through suppressing glucose-6-phosphatase [10]. It is also supposed that over expression inhibits PEPCK gene which encodes phosphoenolpyruvate carboxykinase- an enzyme which is used in the pathway of gluconeogenesis [11], and when over expressed in vitro in presence of high insulin and high glucose in 3T3-L1 adipocytes, there was less amount of glucose uptake probably through disturbing AKT phosphorylation [11]. Increased amount of blood glucose causes inappropriate cell signaling via triggering and disturbing multiple inflammatory and fibrotic pathways in cells of vessels which cause to increase cardiac risks like coronary artery disease, atherosclerosis, stroke and hypertension. Micro RNAs like miR-29, miR-30 and miR-21 are responsible to regulate the fibrotic genes such as collagens and connecting tissue growth factor (CTGF) which leads to fibrosis which is related to heart failure and myocardial infarction [4]. Collagen and VEGF are known targets of MiR-29 [12].When clinical importance of DM related micro RNAs was investigated in the serum of 3 groups, the study showed differential expression of microRNAs when compared for newly diagnosed diabetic group with pre-diabetic and suspected diabetic, however no marked difference was noted among pre-diabetic and suspected diabetic groups in which MiR-29a was also destructed in type 1 diabetes was significantly different in diseased and non-diseased [13]. Years ago the onset and diagnosis of the diabetes the MiRNAs of plasma level changes and can differentiate the patients with a higher chance of having diabetes from healthy measures [14] The whole data suggested the involvement of micro RNA 29 family in pathophysiology of diabetes mellitus. The objective of this study was to compare the expression of micro RNA-29 family in non-diabetic adults of diabetic and non-diabetic parents, so that it may be used as bio marker for early detection of diabetes in non-diabetic adults.

## Main text

### Material and methods

#### Study design and duration

This was a cross-sectional study conducted from January 2019 to January 2021.

#### Sample size calculation

Using Lahr’s theorem from NCSS PASS software, with 95% confident interval and 80% power of the test, the sample size came to be 25 for each group. Sampling technique was purposive convenient type.

#### Participants

Participants were healthy non-diabetic workers and students of Dow University of Health Sciences, Karachi, Pakistan. Participants having age between 18 to 30 years were included after testing for HbA1c according to WHO criteria. While those having any metabolic disorder or HbA1c in rages of pre-diabetes were excluded. Informed written consent was taken from the participants. 50 blood samples were obtained. 25 from each category i.e. with and without parental history of type 2 diabetes mellitus.

#### Ethical approval

The study was approved by institutional review board (IRB) of Dow University of Health Sciences, Karachi, Pakistan (IRB letter no. 2018/1045).

#### Sample preparation

3 ml blood were collected from participants. Consent was taken from every patient and informed about the study. Sample was centrifuged at 6500 rmp for 5 min and plasma was separated and stored at -80 for further process.

#### Extraction and quantification of miRNA

Micro RNA was extracted from samples by using a *mir*Vana® micro RNA isolation kit (Ambion, USA) according to manufacturer’s guidelines.

Extracted micro RNA was then used to form cDNA separately for microRNA 29-a, 29-b, 29c and internal control i.e. U6 using specific RT primers. The synthesized cDNA was used for qRT-PCR by using Maxima SYBR green qPCR master mix (ThermoScientific, USA) according to manufacturer’s instructions and expression of MiR-29a, b and c of samples were normalized to U6. Total 25 µl of reaction volume was attained by adding 12.5 µl of SYBR green master mix, 2 µl of cDNA along with 9.5 µl of dH_2_O, 0.5 µl (10 pmol/µl) of reverse and forward primers. Each sample was run in triplicate. A comparative delta delta-CT formula was used for comparison of targeted micro RNA. Expression of micro RNA was normalized with SnRNA-u6, and comparative values were expressed as 2^−ΔΔCT^.

#### Statistical analysis

Data were compiled in MS Excel and analyzed in SPSS version 21.0. Descriptive statistics expressed in terms of frequency with percentage for categorical variables such as sex, smoking status, exercise status, family history etc. while mean with standard deviation for continuous variables like age, BMI and micro RNA expression were taken.

Repeated Measures ANOVA was used to compare average expression within three members Correlation coefficients between HbA1C, BMI and fold changes were measured by Spearman’s method. With 95% confidence interval, P value less than 0.05 was considered as significant.

## Results

**Fig. 1:**
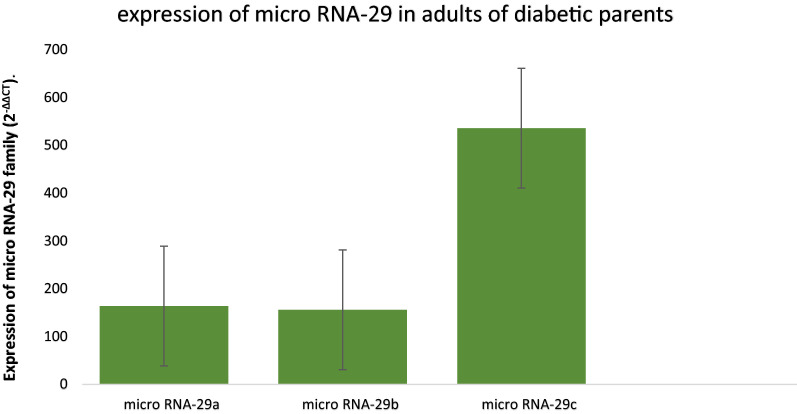
Expressional profile of Micro RNA 29a, 29b and 29c expression in youngsters with parental history of DM

**Fig. 2:**
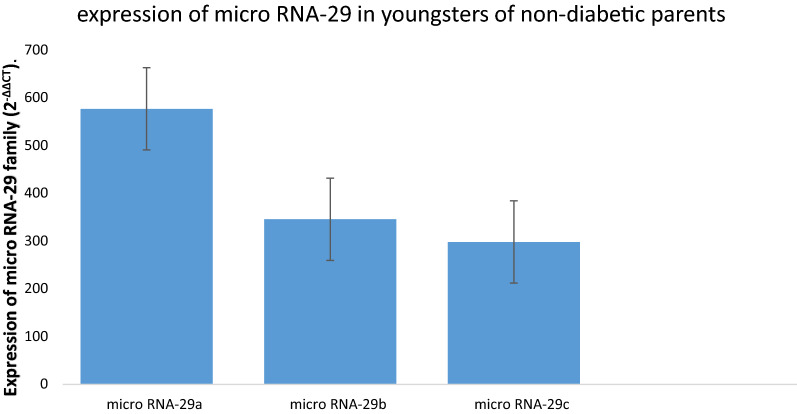
Expressional profile of Micro RNA 29a, 29b and 29c expression in youngsters with no parental history of DM

The demographic data of groups were summarized in Additional file 1: Table S1. When expression of micro RNA was compared within groups separately, it was found that micro RNA 29c was up regulated in youngster with positive family history of diabetes as compared to 29b and 29a (P < 0.001) whereas, no significant difference was found in expression of 29a and 29b in them (P = 0.299) (Fig. 1). Whereas higher expression of micro RNA 29a was found as compared to 29b and 29c in youngsters who had no family history of diabetes (P ≤ 0.001). While no significant differential expression was found between 29b and 29c (P = 0.749) (Fig. 2).

**Fig. 3:**
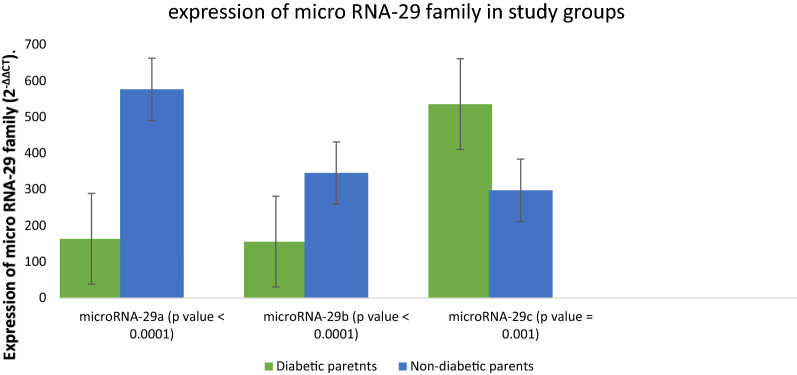
Differential Expression of micro RNAs 29a, 29b and 29c showing significant difference between participants of diabetic and non-diabetic parents

When the expression was compared between the groups with respect to internal control SnRNA-U6, the result showed that micro RNA 29a, 29b and 29c showed significant difference between the both the groups i.e. participants with family history of T2DM and participants without family history of DM (P < 0.001). Additional analysis by post-hoc Tukey test showed marked difference of 29a between both groups (P < 0.0001), in which participants with family history of DM had lower expression of micro RNA-29a as compared to those having no family history of DM. Micro RNA 29b was also found to be down regulated in participants with family history of DM as compared to other group (P < 0.0001). However, Micro RNA 29c was found to be significantly higher in participants with positive family history of DM as compared to those without family history (P = 0.001) (Fig. 3). The fold change of micro RNA of these patients was then compared with different variables like BMI and HbA1c to know the correlation of these variables with expression of microRNA 29 family which revealed no any significant correlation with BMI and HbA1c of participants. [(BMI: 29a, R = -0.093, p = 0.0350; 29b, R = − 0.066, p = 0.508; 29c, R = 0.124, p = 0.214). (HbA1c: 29a, R = − 0.89, p = 0.078; 29b, R = − 0.099, p = 0.023; 29c, R = 0.135, p = 0.322)] (Additional file 2: Figures S1 and S2).

## Discussion

Since the discovery of micro RNA, the initial studies were performed in animal modules to evaluate the functions and pathogenesis of diabetes mellitus related to micro RNA. Multiple studies were carried out to detect and compare the expression of different diabetes related micro RNA in diabetic, pre-diabetic and non-diabetic populations. This study is first of its kind that has evaluated the differential expression of micro RNA-29 in two different non-diabetic population i.e. with and without family history of T2DM to detect possible early changes in micro RNA expression. The effort was made as it can serve as a potential biomarker for early diagnosis of type 2 diabetes mellitus.

This study shows the differential expression of micro RNA-29 family (29a, 29b, 29c) between non-diabetic youngsters with and without family history of DM. This study was however, comprised of only non-diabetic youngsters, but its results correlate with many other studies from past which were performed in different study groups like diabetic, pre-diabetic and non-diabetics.

This research showed micro RNA 29a was down regulated in participants with positive family history of diabetes which is in accordance with many studies; Lin et al. quantified miR-29a and was found down regulated in glomeruli of diabetic mice [15] while another study revealed MiR-29a knockout mice showed higher blood glucose levels when compared with wild type due defect in insulin secretion while insulin production was found to be normal [5]. Satake et al*.* also reported down regulation of micro RNA 29a in plasma of type 1 diabetic patients [16]. Whereas in contrary with our study, Kerolina et al*.* reported the higher expression of micro RNA 29a in serum of newly diagnosed type 2 diabetic patients in order to identify the possible predictors of type 2 diabetes [17]. Dooley et al*.* reported micro RNA 29a as positive regulator of insulin secretion in vivo and here in our study we found down regulation of micro RNA 29a which may lead to decreased insulin secretion in future and cause insulin insensitivity [5].

Here in this study it was found that micro RNA 29b also had low expression which is similar to a study performed on diabetic rats and reported that decreased micro RNA 29 family expression increases the blood glucose levels via interacting negatively with hepatic gluconeogenesis and increase levels of PGC-1ɑ and G6Pase, which are the direct targets of 29 family, which is strongly in favor of this study and lower expression of 29b may cause higher glucose levels in future [10]. Chen et al*.* reported the down regulation of micro RNA 29b in db/db mice which caused increased fibrosis and increased inflammation in the kidney of mice which leads to diabetic nephropathy, which definitely favor these results and also suggests the disturbed level of micro RNA 29 family may lead to diabetic complications [18]. Peng et al*.* also found decreased expression of micro RNA-29b in 83 type 2 diabetic patients [19]. Interestingly, another study revealed an ethnicity specific profile of micro RNA related with T2DM compared with controls between Iraqi and Swedish population also came up with differential expression of micro RNA 29b between the Iraqi and Swedish population which supports this study [20]. However, Kerolina et al. stated the up regulation of 29b in whole blood of 36 T2DM patients along with deregulation of micro RNA-29c [17].

This study also focused on correlation of variables like BMI and HbA1c of diabetic patients with expression of miRNA-29 family but we did not find any correlation of age, BMI and HbA1c with miR-29 expression (Additional file 2: Figures S1a–S2c). While in contrast, different studies have worked on correlation of variables like BMI, FMI, insulin secretion, serum lipids and other metabolites with different micro RNAs in which different correlation was found. Cui et al*.* found a positive correlation between BMI and FMI with miR-222, miR-486, miR-146b, miR-15b, miR-146a, miR-20a, miR-20b, and miR-197 [21]. In another study HbA1c was found to be negatively correlated with miR-126 in 68 T1DM patients [22].

## Conclusion

As micro RNA-29 has proved roll in DM, significant differential expression of micro RNA-29a, 29b and 29c was found between the study groups. As genetic predisposition is one of the cause of type 2 diabetes mellitus, these results suggest possible early genetic changes in non-diabetic adults with positive family history of DM and the same pattern of micro RNA-29 expression is found in patients with diabetes in many studies which are suggestive of involvement of this micro RNA in diabetes and may have potential to serve as early biomarker in type 2 diabetes.

## Limitations

The sample size was small and study population was very concise. Moreover, the micro RNA profile of parents could not be compared with children due to high cost and inconvenience.

## Supplementary Information

**Additional file 1: Table S1.** Summary of demographic profile of participants of the study (n = 50).

**Additional file 2: Figure S1**. a b and c shows no correlation of micro RNA-29a, 29b and 29c with HbA1c of participants. (n = 50). **Figure S2.** a b and c shows no any correlation of micro RNA-29a, 29b and 29c with BMI of participants (n = 50).

## Data Availability

All data has been included in the study however it is available with the corresponding author and may be provided on request.

## Abbreviations

DM: Diabetes mellitus
MiRNA: Micro ribose nucleic acid
VEGF: Vascular endothelial growth factor
