# Supplementary material for: Differential expression of micro RNA-29 family in non-diabetic adults of diabetic and non-diabetic parents
Source: BMC Res Notes. 2021 Jul 28;14:294. doi: 10.1186/s13104-021-05703-8 (PMC8317273; doi:10.1186/s13104-021-05703-8)
Supplement: Supplementary file 1 — Additional file 1: Table S1. Summary of demographic profile of participants of the study (n = 50). [file 13104_2021_5703_MOESM1_ESM.pdf]

Variables		Family History of DM		NO Family History of DM	
Age		26.4	±3.7	25.8	±4.3
Gender	male	16	53.33%	14	46.66%
	female	9	45%	11	55%
BMI		28.96	±3.72	28.62	±3.89
exercise	yes	16	51.61%	15	48.39%
	no	11	57.89%	8	42.11%
smoking	yes	3	50%	3	50%
	no	22	50%	22	50%

Table 1. Summary of demographic profile of participants of the study (n= 50)
