# Supplementary material for: Differential expression of micro RNA-29 family in non-diabetic adults of diabetic and non-diabetic parents
Source: BMC Res Notes. 2021 Jul 28;14:294. doi: 10.1186/s13104-021-05703-8 (PMC8317273; doi:10.1186/s13104-021-05703-8)
Supplement: Supplementary file 2 — Additional file 2: Figure S1. a b and c shows no correlation of micro RNA-29a, 29b and 29c with HbA1c of participants. (n = 50). Figure S2. a b and c shows no any correlation of micro RNA-29a, 29b and 29c with BMI of participants (n = 50). [file 13104_2021_5703_MOESM2_ESM.pdf]

Additional File 02:

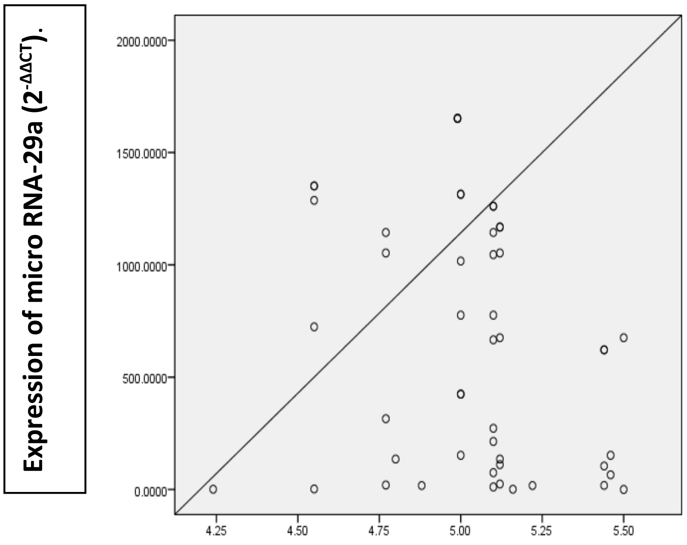

Fig S1a HbA1c (%)

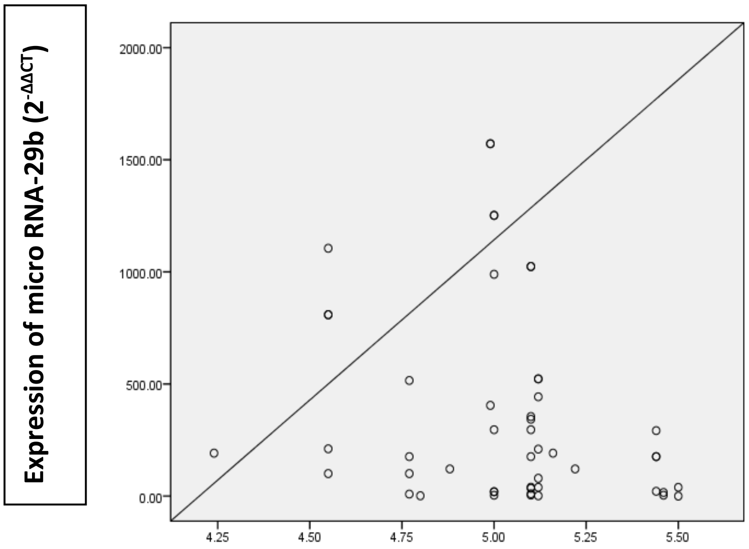

Fig S1b HbA1c (%)

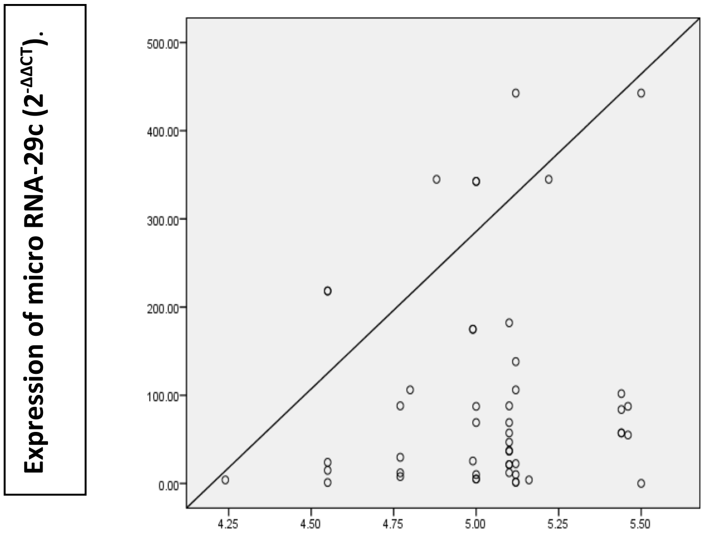

Fig S1c: HbA1c (%)

Fig S1: a b and c shows no correlation of micro RNA-29a, 29b and 29c with HbA1c of participants. (n=50)

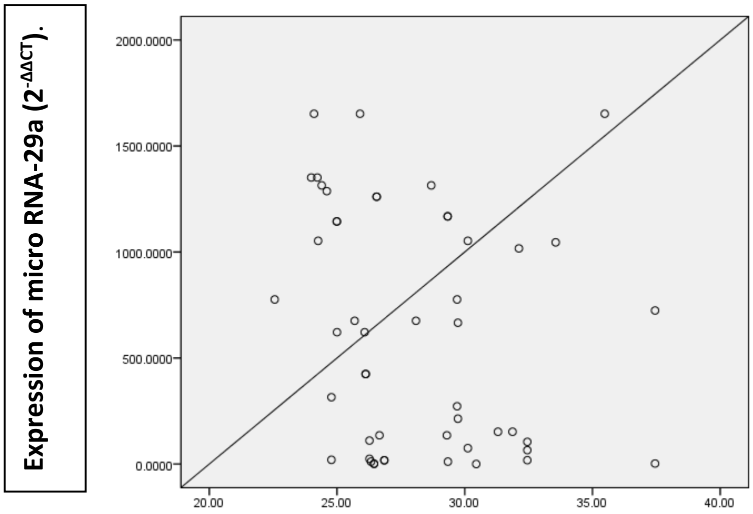

Fig S2a: BMI (Kg/m^2)

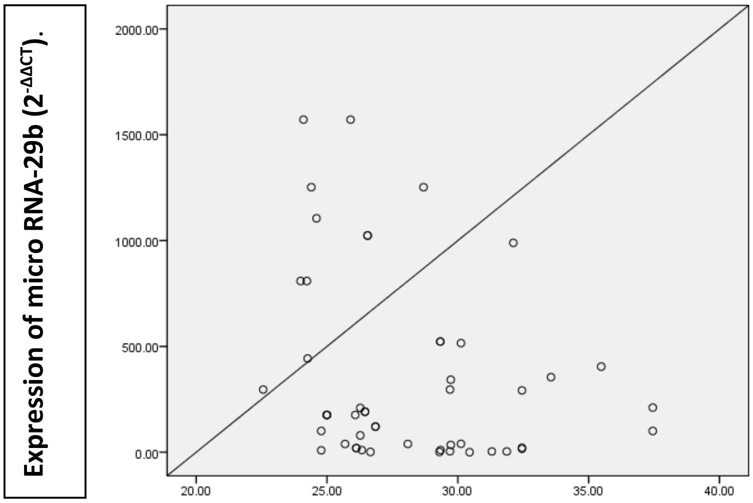

Fig S2b: BMI (Kg/m^2)

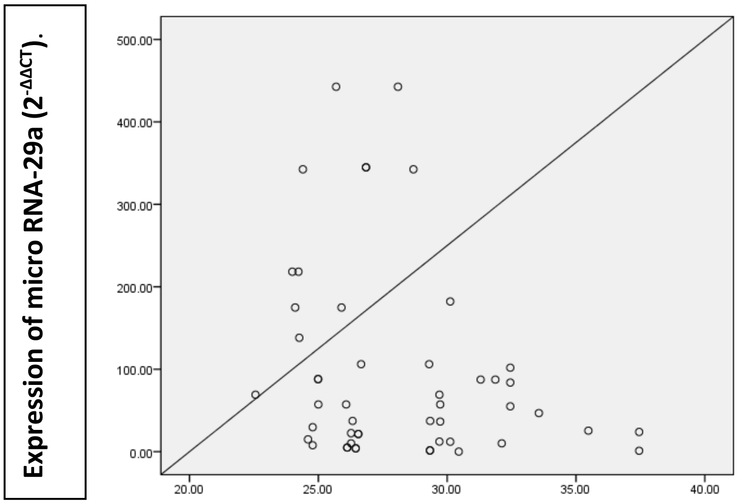

Fig S2c BMI (Kg/m^2)

Fig S2: a b and c shows no any correlation of micro RNA-29a, 29b and 29c with BMI of participants. (n=50)
